# Energy landscapes from cryo-EM snapshots: a benchmarking study

**DOI:** 10.1038/s41598-023-28401-w

**Published:** 2023-01-25

**Authors:** Raison Dsouza, Ghoncheh Mashayekhi, Roshanak Etemadpour, Peter Schwander, Abbas Ourmazd

**Affiliations:** University of Wisconsin Milwaukee, 3135 N. Maryland Ave, Milwaukee, WI 53211 USA

**Keywords:** Computational biophysics, Computational biology and bioinformatics, Computational models

## Abstract

Biomolecules undergo continuous conformational motions, a subset of which are functionally relevant. Understanding, and ultimately controlling biomolecular function are predicated on the ability to map continuous conformational motions, and identify the functionally relevant conformational trajectories. For equilibrium and near-equilibrium processes, function proceeds along minimum-energy pathways on one or more energy landscapes, because higher-energy conformations are only weakly occupied. With the growing interest in identifying functional trajectories, the need for reliable mapping of energy landscapes has become paramount. In response, various data-analytical tools for determining structural variability are emerging. A key question concerns the veracity with which each data-analytical tool can extract functionally relevant conformational trajectories from a collection of single-particle cryo-EM snapshots. Using synthetic data as an independently known ground truth, we benchmark the ability of four leading algorithms to determine biomolecular energy landscapes and identify the functionally relevant conformational paths on these landscapes. Such benchmarking is essential for systematic progress toward atomic-level movies of continuous biomolecular function.

## Introduction

Biomolecular machines have evolved to perform specific tasks through a concerted sequence of conformational motions. There is growing recognition that such motions involve continuous conformational changes, rather than jumps between a small number of discrete states^1,2^. Apart from disordered proteins, conformational continua span a spectrum of different energies. In thermal equilibrium, the probability of a conformational state being occupied is determined by the Boltzmann factor, which drops exponentially with increasing energy.

Conformational motions of proteins can thus be represented as low-lying (and thus strongly occupied) pathways on one or more energy landscapes (EL)^3,4^. In principle, an unlimited number of conformational paths connect a “start” conformation A to an “end” conformation B. However, most such paths include high-energy states, which are sparsely populated under biologically relevant conditions. Due to the exponential nature of the Boltzmann inverse relationship between energy and occupation probability^5^, lowest-energy conformational paths contribute maximally to function.

The growing recognition of the importance of energy landscapes for discerning function has spawned an increasing number of sophisticated algorithms capable of mapping continuous conformational motions. Using a synthetic dataset of cryo-EM snapshots with known ground truth energy landscape, we compare the performance of four leading algorithms, specifically Relion Multibody^6^, CryoSPARC 3DVA^7^, Manifold-EM^8^, and CryoDRGN VAE^9^, in faithfully extracting the energy landscape from snapshots. We benchmark the performance of each algorithmic approach in terms of the accuracy with which the correct energy landscape is recovered from the data.

To date, no comparative benchmarking study of the strengths and weaknesses of different data-analytical approaches for analyzing continuous conformations has been reported. The lack of comparative benchmarks hampers the assessment of the usefulness and reliability of different algorithmic tools in extracting information from experimental data.

In this paper, we use a synthetic dataset generated with an a priori known “ground-truth” energy landscape to benchmark, in silico, the above leading algorithms for conformational analysis of cryo-EM data. Specifically, we quantify each method’s ability to: (a) Recover the correct energy landscape from synthetic cryo-EM datasets; (b) Reveal the functionally important conformational degrees of freedom; and (c) Identify the functionally relevant conformational paths on these landscapes. Although the nature and number of potentially useful algorithms are currently evolving, the four selected approaches represent the state of the art in mapping continuous conformational motions from ensembles of single-particle cryo-EM snapshots.

The primary goals of this paper are thus twofold:

(i) Benchmark the performance of the four leading algorithms listed above in faithfully extracting conformational energy landscapes from synthetic cryo-EM snapshots, and
(ii) Provide a well-characterized synthetic cryo-EM dataset suitable for comparative benchmarking, in order to facilitate the development of more effective data-analytical tools capable of identifying functionally relevant conformational landscapes and motions.

**Figure 1:**
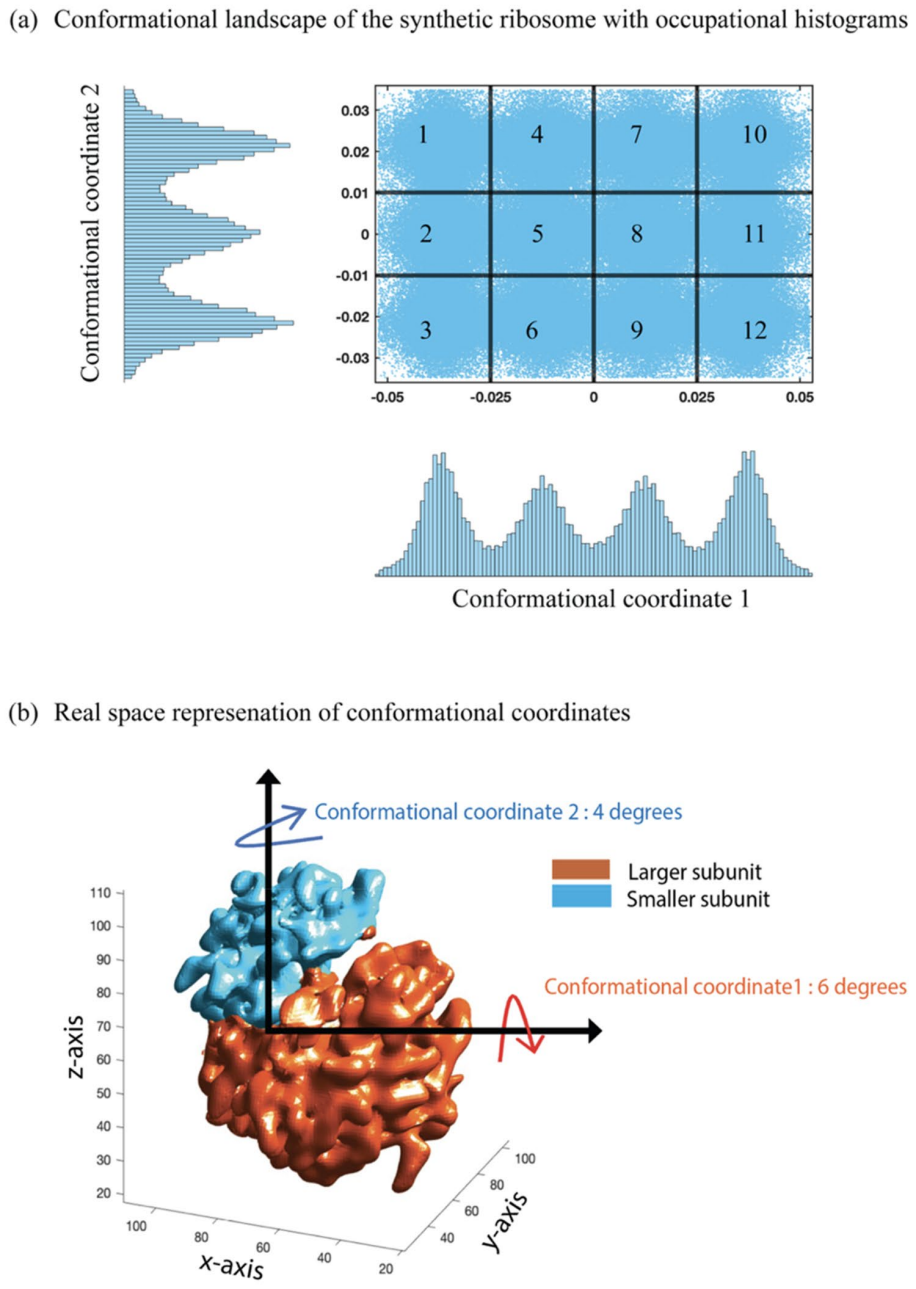
(**a**) The conformational landscape of the synthetic ribosome model along two conformational coordinates containing twelve wells (labelled 1 to 12) of uneven depths. The depths are reflected in the histogram along the two axes. (**b**) Real space representation of the cryo-EM density of the synthetic ribosome model indicating the two conformational directions.

The synthetic dataset of three million cryo-EM snapshots (Signal-to-Noise Ratio SNR = 1) stems from a ribosome-like object with two conformational degrees of freedom, with an underlying energy landscape of 12 energy minima of various depths arranged on a 3 × 4 grid (Fig. 1a). The distribution of points (each representing a single snapshot) is determined by the underlying energy landscape. The landscape is spanned by two conformational degrees of freedom, specifically the rotations of the small subunit (SSU) about two axes named conformational coordinates 1 and 2. The SSU of the ribosome-like object is permitted to rotate in a ratchet-like manner about two mutually orthogonal axes, with the large subunit (LSU) fixed (Fig. 1b).

The performance of each of the four data-analytical approaches is quantified in terms of the fidelity of the energy landscape recovered from the synthetic snapshots. This fidelity is quantified in terms of “Recall” and “Accuracy”, defined in terms of intrinsic distances between snapshots calculated using the Euclidean distance metric. (For details see “Methods” section entitled Accuracy metric).

## Results

Each of the four data-analytical software tools listed above was applied to the synthetic dataset described above. The ground-truth snapshot orientations were provided to each algorithm to focus the study on the ability of the four different algorithms to extract conformational information. In each case, the top two (i.e. the most “powerful”) conformational coordinates were examined. We assess the performance of each algorithm in terms of its ability to classify snapshots correctly into the 12 ground truth energy minima. As outlined below, this approach allows us to quantify algorithmic fidelity in terms of well-known metrics from classification.

### Accuracy of extracted energy landscapes

We use “Recall”^10^ defined as

$$\mathrm{Recall }\,\left(\mathrm{n}\right)=\frac{\mathrm{TP}\,\left(\mathrm{n}\right)}{\mathrm{P }\,\left(\mathrm{n}\right)},$$

where P(n) is the positive class (snapshots belonging to ground truth energy minimum ‘n’), and TP(n) the true positives i.e. snapshots correctly assigned by the algorithm to the energy minimum ‘n’. This allows us to compute a recall value for each of the twelve energy minima. (See SI Tables 3–6).

**Figure 2:**
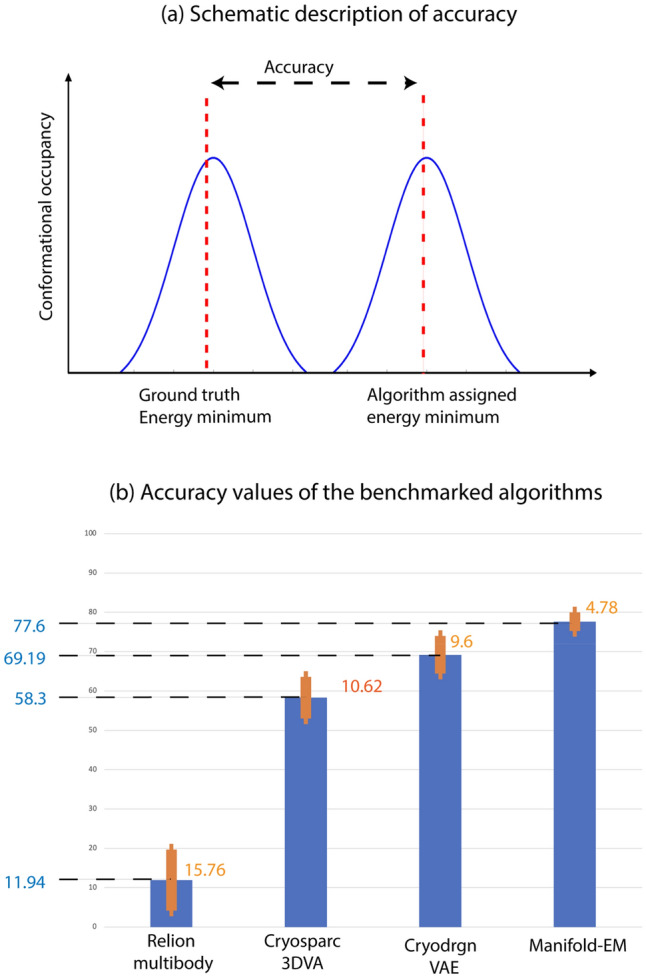
(**a**) A pictorial description of the Accuracy metric calculation in context of energy landscape benchmarking. (**b**) Bar charts showing the accuracy values obtained for each of the four methods.

The average of Recall values, known as Balanced Accuracy^11^ or simply Accuracy, allows us to assign a quantitative score to each algorithm’s ability to accurately extract the energy landscape underlying the synthetic data. (See “Methods” section entitled Accuracy metric). Essentially, one tracks the region of snapshots around the energy minimum in the input landscape and asks, “To what extent is each energy minimum obtained by each algorithm deformed from its original ground-truth shape?” We quantify the extent of this distortion in terms of the Euclidean metric (L2-norm) to calculate a well-defined region of conformational similarity in both the input and output (Fig. 2a,b). The accuracy of each method is given in Table 1.

**Table 1:** Statistics of the Recall metric for the benchmarked algorithms.

Software tools	Accuracy (average recall) (%)	Standard deviation of recall (%)
Relion multibody	17.54	14.35
CryoSPARC 3DVA	51.3	18.63
CryoDRGN	61.2	9.6
Manifold-EM	77.6	4.79

We now summarize the outcome of our benchmarking study. Accuracy is calculated by averaging the Recall values over the 12 energy minima. Relion Multibody on average assigns only 17.54 ± 14.4% of the points to the correct region in the ground-truth energy landscape (Fig. 2a,b). The cryoSPARC 3DVA algorithm achieves an accuracy score of 51.3 ± 18.6%. cryoDRGN variational autoencoder assigns snapshots with an accuracy of 61.2 ± 9.6%. The Manifold-EM algorithm correctly assigns 77.6 ± 4.8% of the snapshots on average over the entire ground truth energy landscape.

### Occupancy maps and energy landscapes

**Figure 3:**
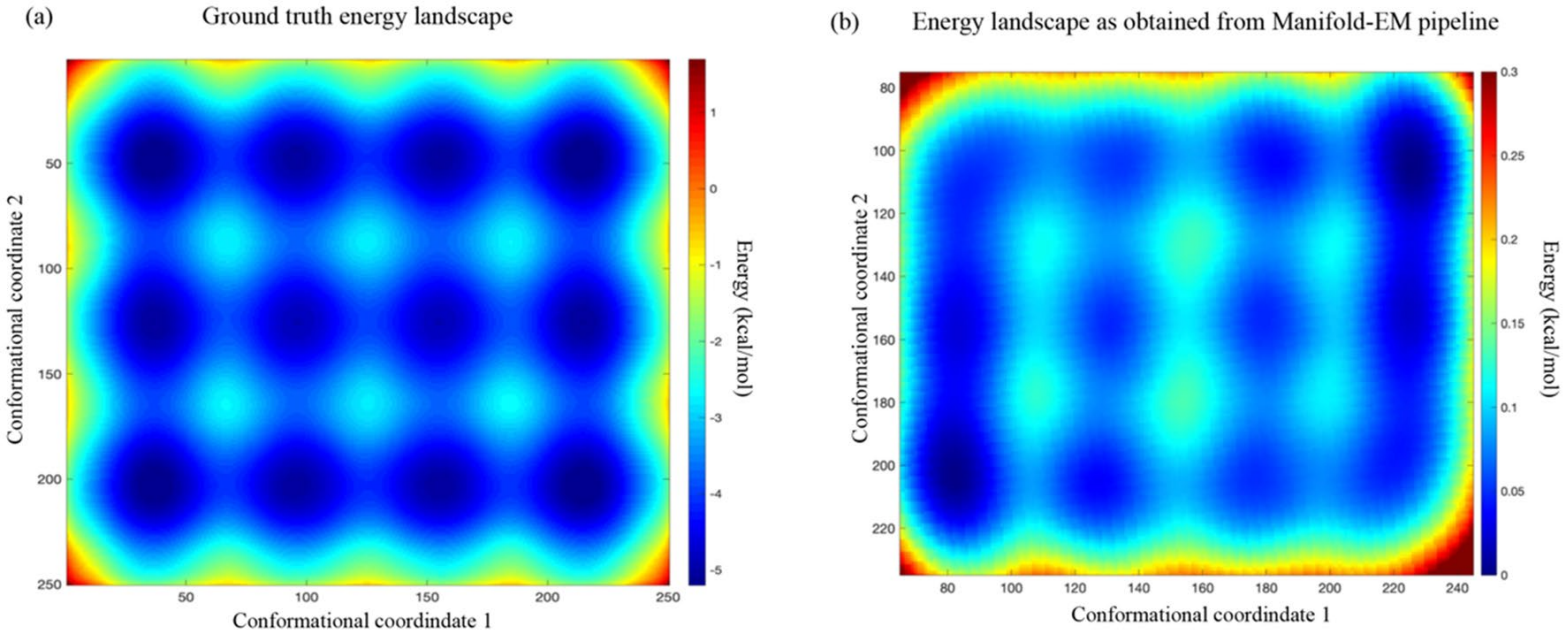
(**a**) Ground-truth energy landscape. (**b**) Energy landscape obtained by the data-analytical pipeline implemented in Manifold-EM.

The occupation probability of a conformational state is determined by the energy of the state via the Boltzmann factor. An occupancy map (defined in^12^) is simply an alternative representation of the conformational energy landscape of the ribosome-like object. Figure 3a shows the energy landscape for the continuous conformational landscape of the synthetic ribosome.

To date, Manifold-EM is the only method that can calculate the energy landscapes of the conformational occupancy. The corresponding energy landscape obtained from the Manifold-EM data analytical pipeline is shown in Fig. 3b. It is evident that the energy landscape obtained by Manifold-EM closely resembles the ground-truth energy landscape. This includes the high occupancy regions or, equivalently, the twelve energy minima (Fig. 3b), while preserving the modulated features (i.e. the variation in depths of the individual energy minima) across the ground truth conformational coordinates (Fig. 3a). The other benchmarked algorithms have not yet developed a way to extract thermodynamic quantities like occupancy probability or free energy. It is evident that the energy landscape obtained by Manifold-EM.

### Visualizing the energy landscape

**Figure 4:**
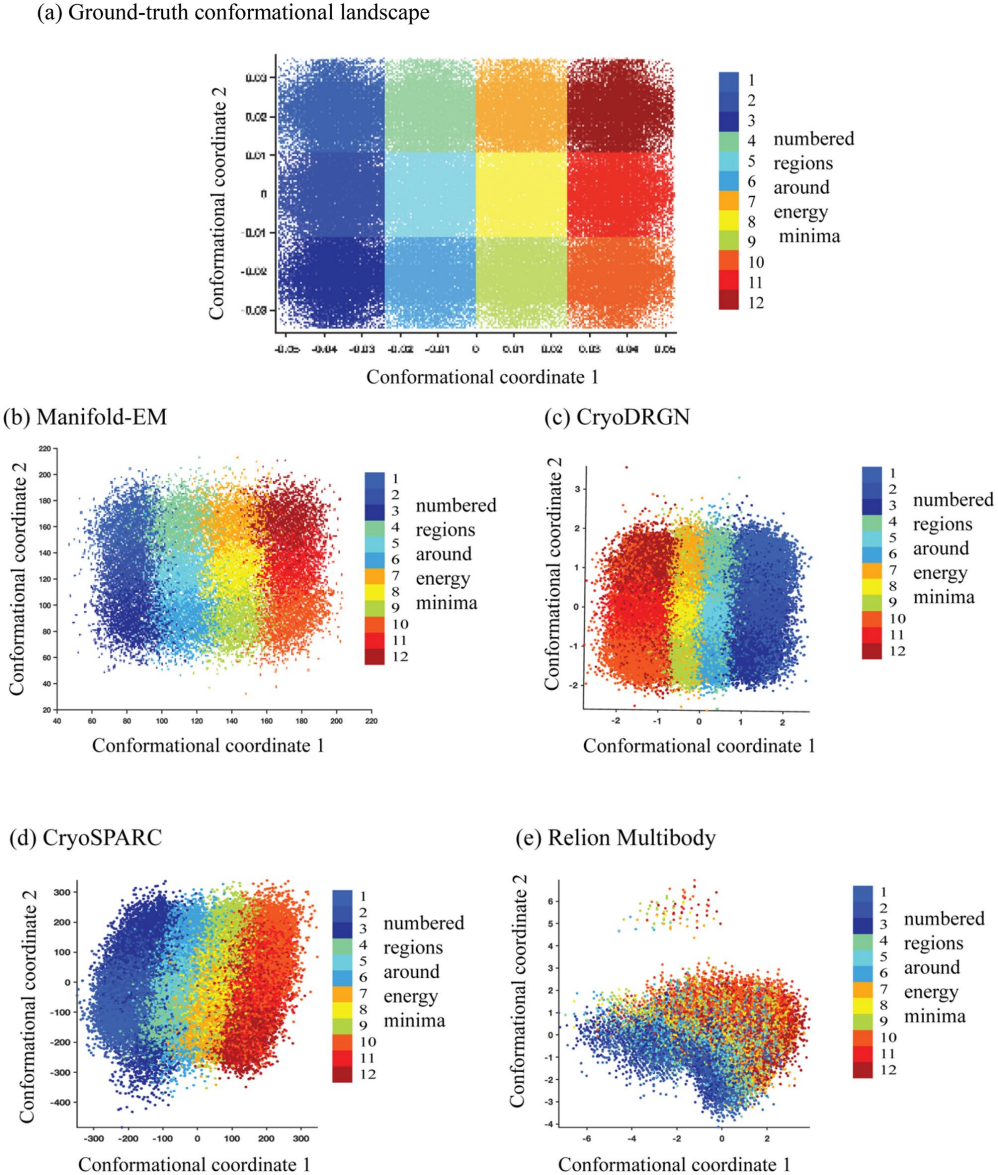
Ground-truth and output conformational landscapes. (**a**) The lineage colored according to the ground-truth location. (**b–e**) The lineage obtained from Manifold-EM, CryoDRGN, CryoSPARC and Relion Multibody, respectively, using panel (**a**) as reference.

The performance of the benchmarked algorithms can be validated by using methods from data-visualization. We segment the ground truth energy minima using a multi-color approach. This procedure is known as ‘data lineage’^13^. Each energy minimum has been assigned a unique color to identify each energy minimum. (Fig. 4a). Essentially, lineage uses the ground truth labels for each single-particle snapshot in the energy landscape to identify its position in the output of each of the data-analytical tools (Fig. 4b–e). The region surrounding each of the 12-energy minima is rendered in a different color to elucidate the extent to which snapshots stemming from adjacent regions of the ground truth energy landscape are correctly assigned by each of the four data-analytical techniques.

As shown in Fig. 4b, the overall ground-truth topology is recovered by Manifold-EM. It is evident from Fig. 4c–e that the alternative approaches, viz. Relion, cryoSPARC, and cryoDRGN severely distort the energy landscape, intermixing points stemming from different energy minima.

## Discussion

Inferring biological function from biomolecular structure is a paramount goal of structural biology. The results presented here highlight the importance of basing functional inference on energy landscapes and conformational coordinates derived from the data. To this end, we have developed and tested a rigorous approach to benchmarking the performance of four leading data-analytical algorithms in faithfully extracting energy landscapes underlying a collection of synthetic snapshots. This assessment is based on the accuracy with which individual snapshots are placed on the output energy landscape as compared with the independently known ground-truth distribution. Using a complex synthetic model, Manifold-EM, accurately recovers the energy landscape.

In view of the importance of inferring function from structure, we anticipate an increasing spectrum of techniques for mapping energy landscapes. The benchmarking data we have used will facilitates rigorous assessments of the efficacy and reliability of current and future tools for determining biomolecular structure and function. Our approach thus offers a rigorous means for measuring and improving the performance of data-analytical approaches capable of extracting energy landscapes from cryo-EM datasets.

The study design, including the synthetic ribosome-like object, was chosen to mimic conditions typically encountered in conformational motions in biological macromolecules. Of course, any benchmarking exercise pertains to a particular time point. Consistent with the rapid progress in cryo-EM single-particle imaging, data-analytical software tools are evolving apace^14–18^. Our approach offers a quantitative method for assessing and guiding this rapidly progressing field.

The recall and accuracy metric are powerful tools for comparing the different algorithms, as it allows the study of energy regions without prior assumptions about the shape and distribution of the energy landscapes. Despite differences in the formulation of the different algorithms, the accuracy-based score can be consistently applied to present and emerging algorithms. Currently, Manifold-EM represents the most reliable means for extracting energy landscapes and conformational coordinates from single-particle cryo-EM images. We offer the synthetic data used in the present study as a canonical test vehicle for developing powerful algorithms for extracting reliable structural and functional information from cryo-EM data.

## Methods

### Accuracy metric

Finding an appropriate metric for extracting conformational landscapes from cryo-EM data is a challenging task, if only because the metric must quantify the distortion caused by the algorithmic approach. We tackle this problem by using the quantitative metrics Recall and Accuracy^11^, in order to quantify the ability of each of the four algorithms to correctly assign each snapshot to one of 12 classes. The Recall metric was implemented as follows:

1. The center of each energy minimum was obtained by calculating the ‘mean’ of all the particles corresponding to that minimum region (for all 12 regions).
2. Compute the distance matrix (squared Euclidean) using the 12 centers
3. Using the distance of each particle from the corresponding center (Fig. 4a), bin the particles into each minimum based on the shortest distance to the center.
4. This procedure assigns particles to a label from 1 to 12.

The Accuracy^11^ of the benchmarked method is given by:

$$\mathrm{Accuracy }=\frac{{\sum }_{\mathrm{n}}\mathrm{Recall}\,(\mathrm{n})}{\mathrm{n}},$$

where n is the number of energy minima (in our case, 12) and the numerator is the sum of the Recall metric calculated for each energy minima.

Recall ranges from 0 to 1 (as evident from the definition), where a value of 0 implies no snapshot is correctly assigned to that energy minima and a value of 1 when all snapshots belonging to the energy minima are correctly allocated. The accuracy of each algorithm is the average recall metric for assignment into all 12 minima.

## Supplementary Information

Supplementary Information.

## Data Availability

The datasets used and/or analyzed, and the code developed in the course of the current study are available from the corresponding author on reasonable request.
