# Supplementary material for: Energy landscapes from cryo-EM snapshots: a benchmarking study
Source: Sci Rep. 2023 Jan 25;13:1372. doi: 10.1038/s41598-023-28401-w (PMC9876912; doi:10.1038/s41598-023-28401-w)
Supplement: Supplementary file 1 — Supplementary Information. [file 41598_2023_28401_MOESM1_ESM.docx]

**­Energy Landscapes from Cryo-EM Snapshots:**

**A Benchmarking Study**

**SUPPLEMENTARY INFORMATION**

Raison Dsouza, Ghoncheh Mashayekhi, Roshanak Etemadpour, Peter Schwander,

Abbas Ourmazd^*^

University of Wisconsin Milwaukee, 3135 N. Maryland Ave, Milwaukee WI 53211, USA

Table of Contents Page Number

[Input data and preprocessing steps 3](#_Toc121992568)

[Conformational energy landscapes 4](#_Toc121992569)

[Patching procedure used in Manifold-EM 5](#_Toc121992570)

[Computational resources 6](#_Toc121992571)

[Supplementary Tables 6](#_Toc121992572)

[Supplementary References 13](#_Toc121992573)

[Supplementary Figures 14](#_Toc121992574)

**Supplementary Tables 1-6**

**Supplementary Figures 1-6**

#

# **Input data and preprocessing steps**

This section describes the generation of the synthetic cryo-EM snapshots and the steps involved in single-particle cryo-EM image preprocessing. A ribosome-like synthetic model with two continuous conformational degrees of freedom entailing rotations of the small subunit about two different axes was simulated and used as the ground truth (Supplementary Figure 1(b)). The 3D density map of an 80S Ribosome at 7Å [1] was used as the source for the data. The ground-truth conformations lie on a two-dimensional flat manifold (Supplementary Figure 1(a)), which corresponds to the rotation of the small subunit with respect to the large subunit. We use 180 projection directions along a great circle, each containing 18,000 single-particle images projected along the x-axis. The images were not subjected to the microscope contrast transfer function. A sample image with and without noise is shown in Supplementary Figures 2(a) and 2(b). Pixel noise was incorporated via a Gaussian model and particle images individually normalized to have unit signal variance.

Synthetic cryo-EM snapshots of different conformations were generated with probabilities reflecting a conformational energy landscape consisting of a rectangular 3x4 array of 12 energy minima of different depths (Supplementary Figure 1(a)). The 3x4 array was selected to reflect a conformational change of 6 degrees along conformational coordinate 1, and 4 degrees along conformational coordinate 2, respectively. The ground-truth energy landscape mimics the biomolecule traversing through stable states and transition states. In this regard, we formulated an occupancy distribution via a potential with pockets to reflect energy minima. Conformational coordinate 1 was assigned four energy minima, while conformational coordinate 2 was assigned three energy minima.

# **Conformational energy landscapes**

The data-analytical tools we use [2-5] utilize machine learning algorithms to determine from the snapshots the number of degrees of freedom exercised by the biomolecule. To assess each algorithm’s ability to capture continuous conformational changes, we examined how the input data are distributed along the two leading eigenvectors commonly calculated in the algorithms used to determine energy landscapes. Binning the data along these eigenvectors allows us to easily visualize the distribution of snapshots as histograms. The histograms of the individual conformational coordinates are shown in Supplementary Figure 3(a-d). The histograms of conformational coordinates essentially point out the occupancy of each region in the energy landscape. For example, in the ground-truth energy landscape, conformational coordinate 1 shows four heavily occupied regions (in energy landscape terms, four minima). Similarly, conformational coordinate 2 shows three conformational maxima, or equivalently, three energy minima.

To determine the relationship between the different eigenvectors obtained from each method, we plot the snapshots as points in a two-dimensional diagram. Such so-called ‘scatter plots’ enable one to visualize the data distribution along different degrees of freedom extracted from the data. Figures 3 (a-d) show the scatter plots along each conformational coordinate with the corresponding histograms for each component (along the margin of each axis). The histograms of conformational coordinates obtained from Manifold-EM (Supplementary Figure 3(a)) show the four energy maxima along coordinate 1 and three maxima along coordinate 2. However, the heights of the peaks along conformational coordinate differ from the ground truth. This leads to some particles being assigned to incorrect energy minima, which results in false negatives in the calculation of the Accuracy score (see Methods). In cryoDRGN (Supplementary Figure 3(b)), the particle distribution has a Gaussian-like distribution along conformational coordinates one and two, which leads to incorrect assignment of particles to different energy minima. This results in an increased standard deviation in the Accuracy scores. In Relion multibody (Supplementary Figure 3(c)), the marginal histograms for the first two principal components are bimodal, and essential features of the ground truth distribution are lost. This causes a drastic reduction in the Accuracy score indicating snapshots are assigned incorrectly. For CryoSPARC 3DVA (Supplementary Figure 3(d)), the histograms show an almost featureless histogram for conformational coordinate 1 and monomodal (bell-shaped) distribution for conformational coordinate 2. The flat distribution essentially misses on the less-pronounced, but potentially important features of the ground-truth energy landscape.

# **Patching procedure used in Manifold-EM**

Manifold-EM [2, 6, 7] uses diffusion map embedding of the snapshots falling into each 180 projection directions along a great circle of molecular orientations to extract the conformational manifold and the set of orthogonal eigenfunctions describing this manifold. In the present study, two eigenfunctions suffice to capture the conformational information. The occupancy map and the corresponding free-energy landscape are deduced from the distribution of points representing conformational states. Since the conformational coordinates in each 2D manifold of each projection direction are sorted according to their eigenvalues, their ranking can change depending on the projection direction (Supplementary Figure 4(a)). Additionally, the conformational changes along each conformational coordinate (CC) acquire arbitrary signs (senses) due to eigen-decomposition. Therefore, to aggregate all the occupancy planes belonging to each projection direction, it is necessary to establish a consistent conformational sense for all projection directions. To achieve this goal, we use an approach exploiting the correlation coefficient between adjacent projection directions.

In the present study, the 2D manifolds obtained using Manifold-EM have a rectangular shape. We take samples of snapshots from each of the four corners of the 2D manifold (Supplementary Figure 4(b)) and calculate the correlation coefficient between the image average from each corner of a given projection direction and four image averages of the adjacent projection direction (Supplementary Figure 5). We use the correlation coefficient to align conformational manifolds from adjacent projection directions (See Supplementary Figure 6). Armed with consistently aligned conformational manifolds from all projection directions, we compile the occupancy map and the corresponding energy landscape.

# **Computational resources**

All computations were performed on these two machines with the following specifications:

1. Intel CPU/Nvidia GPU cluster, 320 CPU cores arranged as 16 nodes, each equipped with two Deca-core E5-2660 V3 “Haswell”/2.6GHz, 128GB RAM; 79872 GPU cores arranged as 16 Tesla K80 Dual GPU cards.
2. Intel Server with dedicated GPU: Intel(R) Xeon(R) Gold 5115 CPU/2.40GHz, 20 cores, 384 GB RAM, NVIDIA Tesla V100, 5120 GPU cores, 640 tensor cores.

# **Supplementary Tables**

| Synthetic Dataset | Number of Particles | Image Size (pixels) | Pixel Size  (Å) | Latent-Space Dimension |
| --- | --- | --- | --- | --- |
|  | 3,240,000 | 124x124 | 2.4 | 2 |

**Supplementary Table 1: Dataset characteristics**

| Method | Parameter | Value |
| --- | --- | --- |
| CryoDRGN | Software version | 1.0 |
|  | Dimension of latent variable | 2 |
|  | Number of training epochs | 25 |
|  | Number of nodes in hidden layers (Encoder & Decoder) | 1024 |
|  | Number of hidden layers  (Encoder & Decoder) | 3 |
| CryoSPARC | Software version | 2.9 |
|  | Number of modes to solve | 3 |
|  | Filter resolution (Å) | 7 |
| Relion Multibody | Software version | 3.1.0 |
|  | Standard deviations for Gaussian prior for rotations (degrees) | 6 |
|  | Standard deviations for Gaussian prior for translations (Å) | 1 |
| Manifold-EM | Software version | 1 |
|  | Gaussian weights | 10 |
|  | Number of nearest neighbors in the graph | 18000 |

**Supplementary Table 2: Parameters used in the individual algorithms for the benchmark**

| **Region in the energy landscape estimated using Relion Multi-body** | **True Positives** | **Recall (%)** |
| --- | --- | --- |
| Energy minimum 1 | 111411 | 38.55 |
| Energy minimum 2 | 11814 | 4.299 |
| Energy minimum 3 | 87906 | 30.48 |
| Energy minimum 4 | 27727 | 10.61 |
| Energy minimum 5 | 8949 | 3.61 |
| Energy minimum 6 | 40624 | 15.48 |
| Energy minimum 7 | 30824 | 11.72 |
| Energy minimum 8 | 7846 | 3.14 |
| Energy minimum 9 | 31640 | 12.04 |
| Energy minimum 10 | 69794 | 23.93 |
| Energy minimum 11 | 26985 | 9.79 |
| Energy minimum 12 | 136530 | 46.94 |

**Supplementary Table 3. Recall scores for each energy minimum region obtained from the Relion Multi-body algorithm.**

| **Region in the energy landscape estimated using cryoSPARC** | **True Positives** | **Recall (%)** |
| --- | --- | --- |
| Energy minimum 1 | 163349 | 70.65 |
| Energy minimum 2 | 75373 | 34.21 |
| Energy minimum 3 | 149773 | 64.87 |
| Energy minimum 4 | 122002 | 58.21 |
| Energy minimum 5 | 51724 | 26.03 |
| Energy minimum 6 | 127377 | 60.53 |
| Energy minimum 7 | 115827 | 55.05 |
| Energy minimum 8 | 47827 | 23.96 |
| Energy minimum 9 | 135398 | 64.37 |
| Energy minimum 10 | 142466 | 61.04 |
| Energy minimum 11 | 53648 | 24.33 |
| Energy minimum 12 | 168327 | 72.29 |

**Supplementary Table 4. Recall scores for each energy minimum region obtained from the CryoSPARC 3DVA algorithm.**

| **Region in the energy landscape estimated using CryoDRGN** | **True Positives** | **Recall (%)** |
| --- | --- | --- |
| Energy minimum 1 | 188659 | 65.27 |
| Energy minimum 2 | 134148 | 48.72 |
| Energy minimum 3 | 198849 | 68.94 |
| Energy minimum 4 | 176817 | 67.66 |
| Energy minimum 5 | 121080 | 48.86 |
| Energy minimum 6 | 174829 | 66.65 |
| Energy minimum 7 | 180603 | 68.67 |
| Energy minimum 8 | 121864 | 48.85 |
| Energy minimum 9 | 178956 | 68.10 |
| Energy minimum 10 | 200691 | 68.82 |
| Energy minimum 11 | 128649 | 46.70 |
| Energy minimum 12 | 195169 | 67.11 |

**Supplementary Table 5. Recall scores for each energy minimum region obtained from the CryoDRGN VAE algorithm.**

| **Region in the energy landscape estimated using Manifold-EM** | **True Positives** | **Recall (%)** |
| --- | --- | --- |
| Energy minimum 1 | 237822 | 82.49 |
| Energy minimum 2 | 201863 | 75.24 |
| Energy minimum 3 | 241547 | 84.16 |
| Energy minimum 4 | 203118 | 76.82 |
| Energy minimum 5 | 175348 | 71.76 |
| Energy minimum 6 | 204082 | 77.00 |
| Energy minimum 7 | 205244 | 77.18 |
| Energy minimum 8 | 177382 | 72.11 |
| Energy minimum 9 | 203160 | 83.26 |
| Energy minimum 10 | 241632 | 83.26 |
| Energy minimum 11 | 200414 | 74.99 |
| Energy minimum 12 | 238875 | 82.90 |

**Supplementary Table 6. Recall scores for each energy minimum region obtained from the Manifold-EM algorithm.**

# **Supplementary References**

1. Frank, J. and R.K. Agrawal, *A ratchet-like inter-subunit reorganization of the ribosome during translocation*, in *Single-Particle Cryo-Electron Microscopy: The Path Toward Atomic Resolution: Selected Papers of Joachim Frank with Commentaries*. 2018, World Scientific. p. 269-273.

2. Dashti, A., et al., *Trajectories of the ribosome as a Brownian nanomachine.* Proc Natl Acad Sci U S A, 2014. **111**(49): p. 17492-7.

3. Zhong, E.D., et al., *CryoDRGN: reconstruction of heterogeneous cryo-EM structures using neural networks.* Nat Methods, 2021. **18**(2): p. 176-185.

4. Punjani, A. and D.J. Fleet, *3D variability analysis: Resolving continuous flexibility and discrete heterogeneity from single particle cryo-EM.* J Struct Biol, 2021. **213**(2): p. 107702.

5. Nakane, T., et al., *Characterisation of molecular motions in cryo-EM single-particle data by multi-body refinement in RELION.* Elife, 2018. **7**.

6. Dashti, A., et al., *Retrieving functional pathways of biomolecules from single-particle snapshots.* Nat Commun, 2020. **11**(1): p. 4734.

7. Ourmazd, A., *Cryo-EM, XFELs and the structure conundrum in structural biology.* Nature methods., 2019. **16**(10): p. 941.

# **Supplementary Figures**


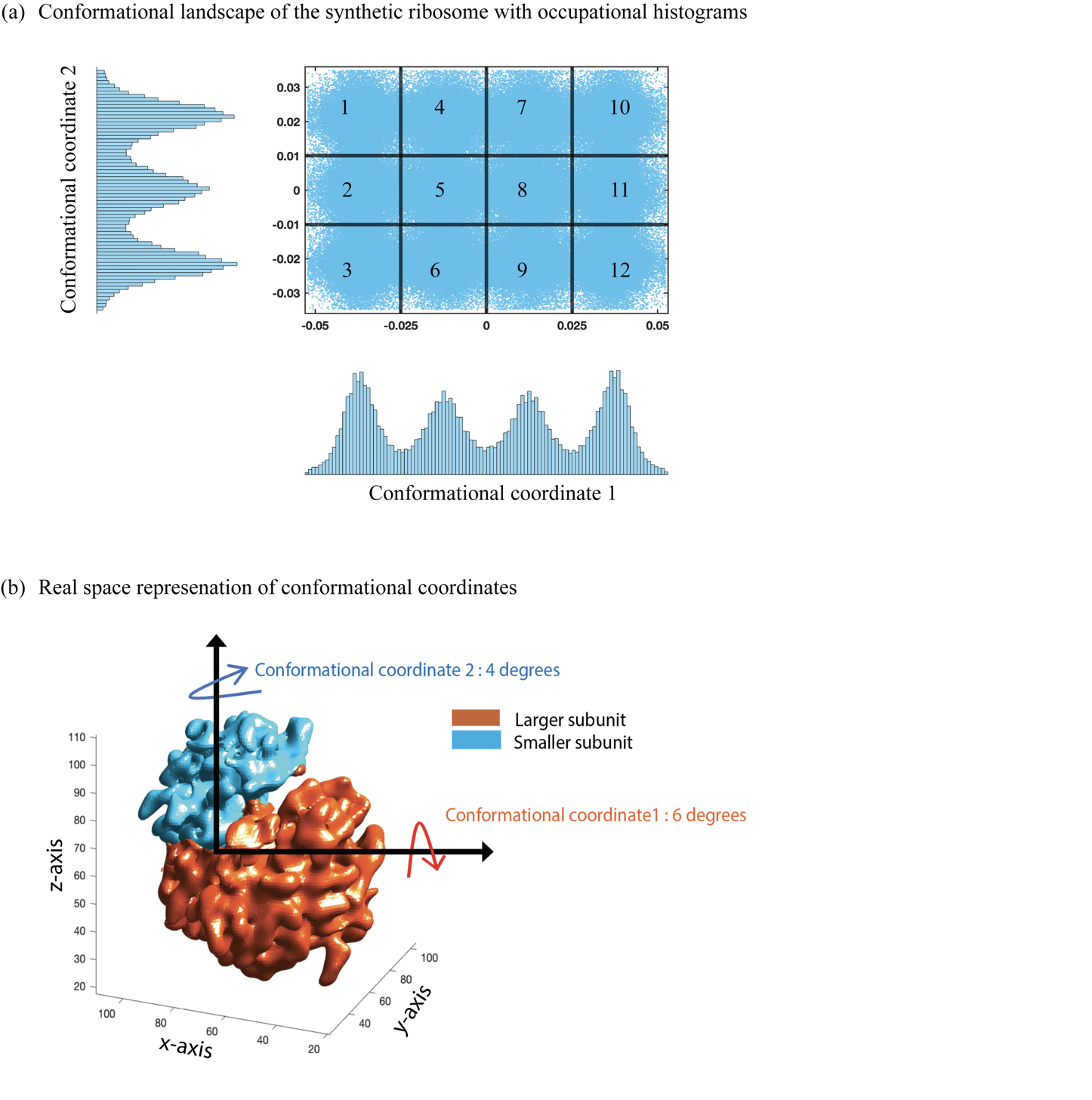


**Supplementary Fig. 1. (a) Ground-truth conformational landscape for the synthetic model with 12 high occupancy regions (labelled 1 to 12), indicated as maxima on the histograms.**

**(b) Sketch of the synthetic model, rotation of the small subunit about the two axes labeled conformational coordinate 1 and 2 respectively. The large subunit (red) is fixed.**

**
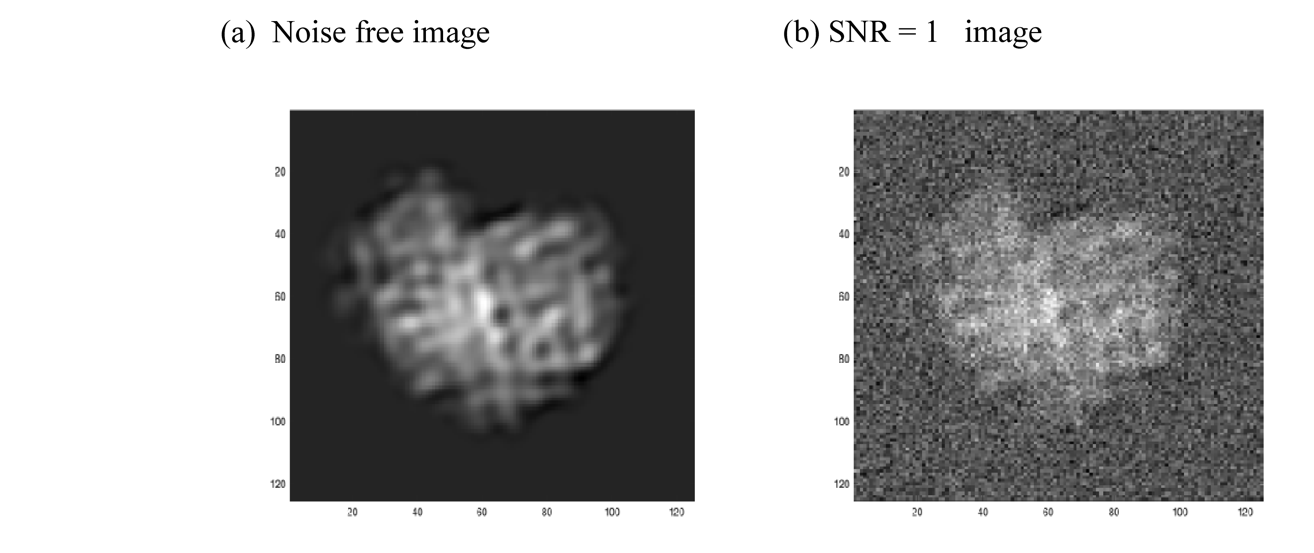
**

**Supplementary Fig. 2. Images of the synthetic data. (a) Without pixel noise. (b) With pixel noise (SNR=1)**

**
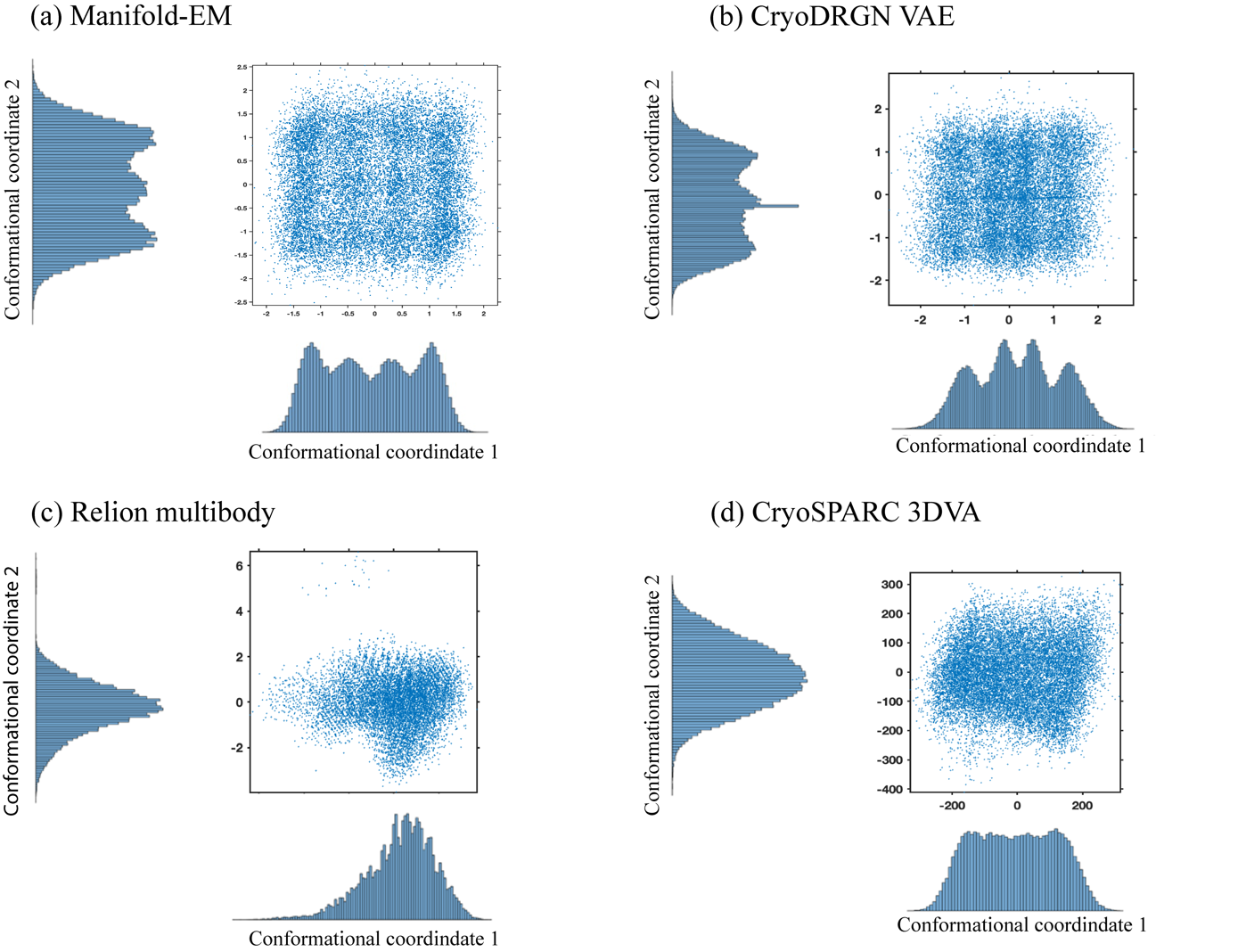
**

**Supplementary Fig. 3. Scatter plots of the two retrieved conformational coordinates obtained from each of the four algorithms. Only 18000 points have been plotted to avoid visual crowding of points. Also shown are the marginal histograms for each case with the full population of points.**

**
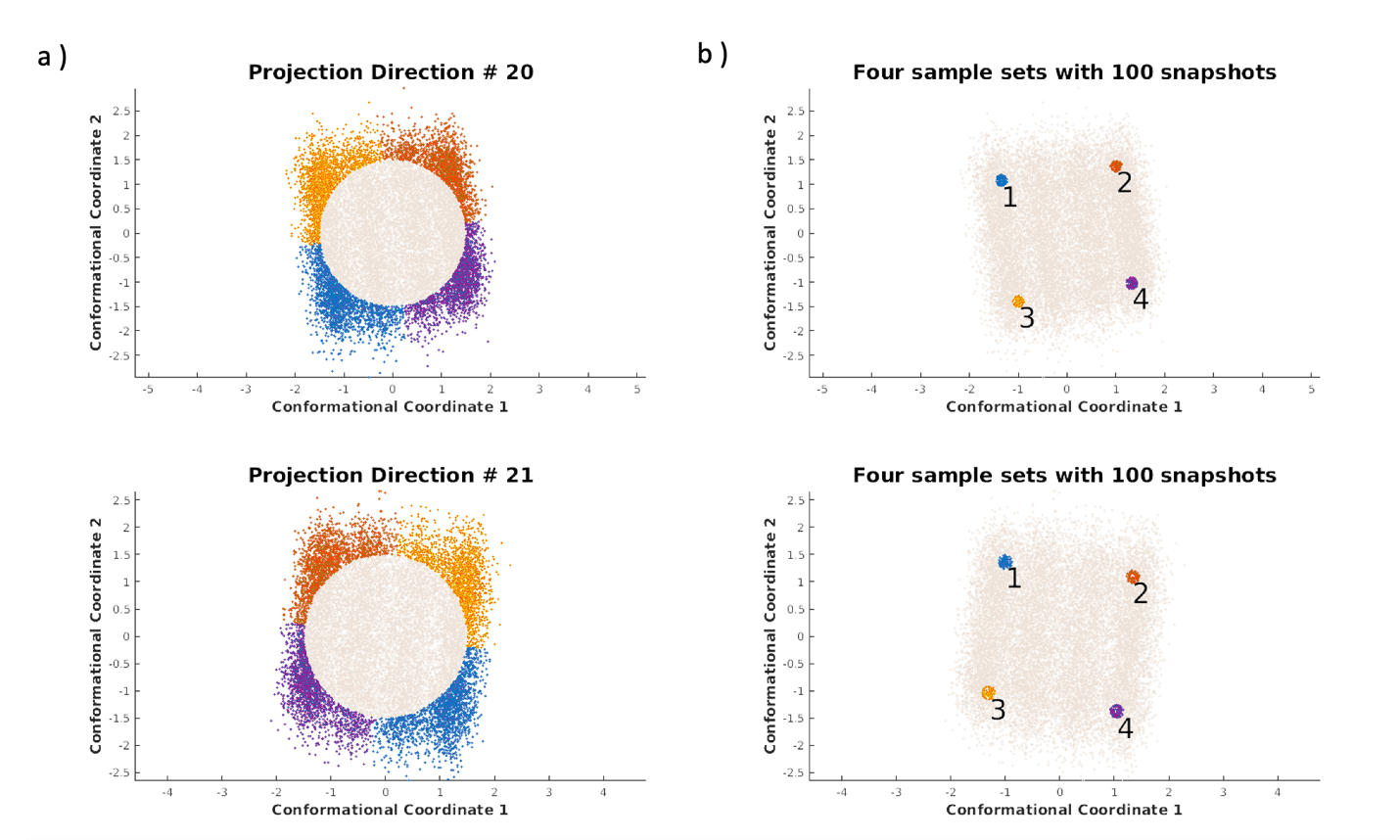
**

**Supplementary Fig. 4.**

**(a) 2D quasi-rectangular conformational planes for two adjacent projection directions (20 and 21). (b) Snapshots from the four corners in the Conformational Coordinates (CC) 1 and 2 plane.**


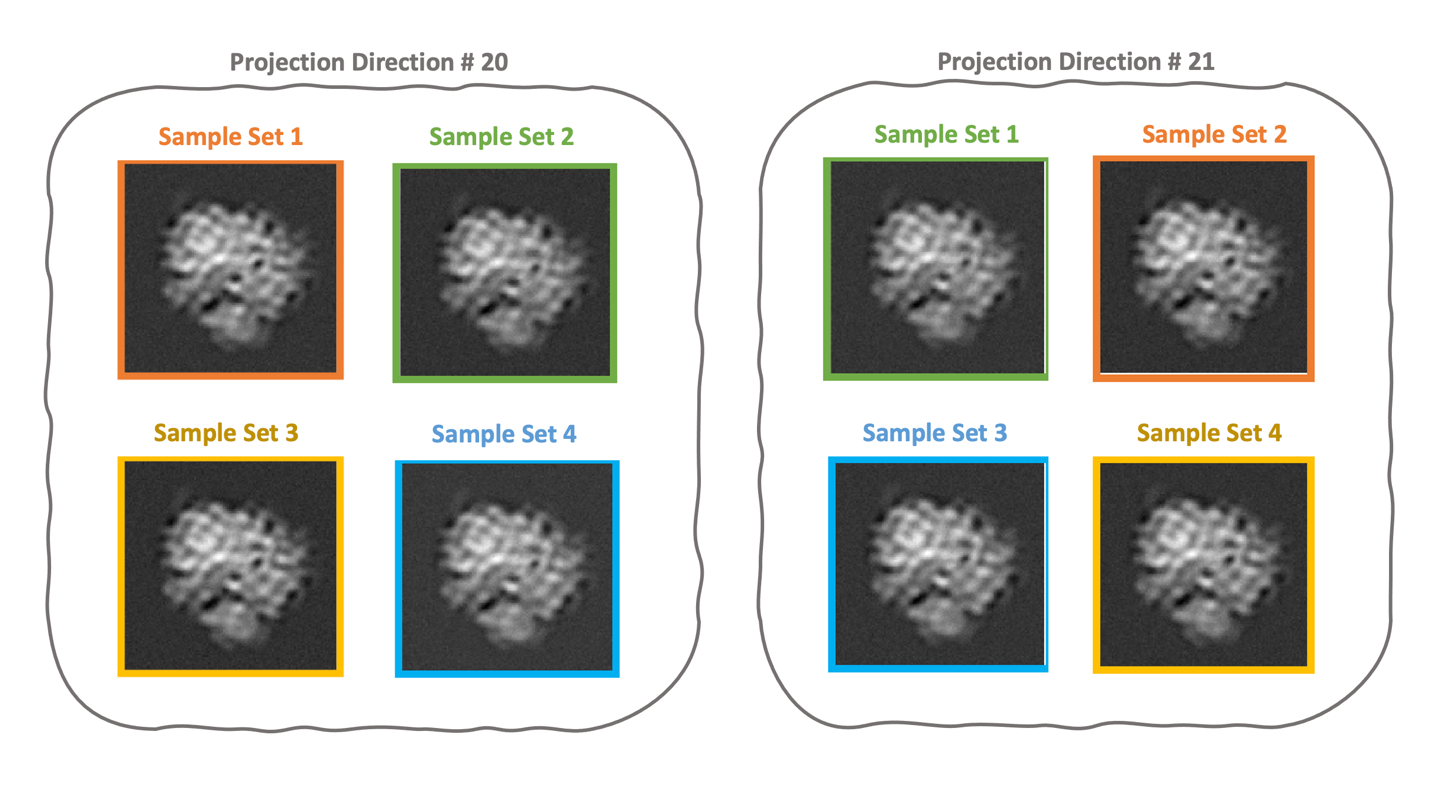


**Supplementary Fig. 5.**

**The image averages of four sample sets of the projection directions 20 and 21. The boxes with similar colors illustrate the most correlated image averages. Here, the order [1,2,3,4] of the projection direction 20 has been transformed to the new order [2,1,4,3] in the following projection direction 21. Hence, based on the model, the sign of the CC1 of projection direction 21 needs to be changed relative to the CC1 of projection direction 20, while the sign of CC2 remains unchanged.**

**
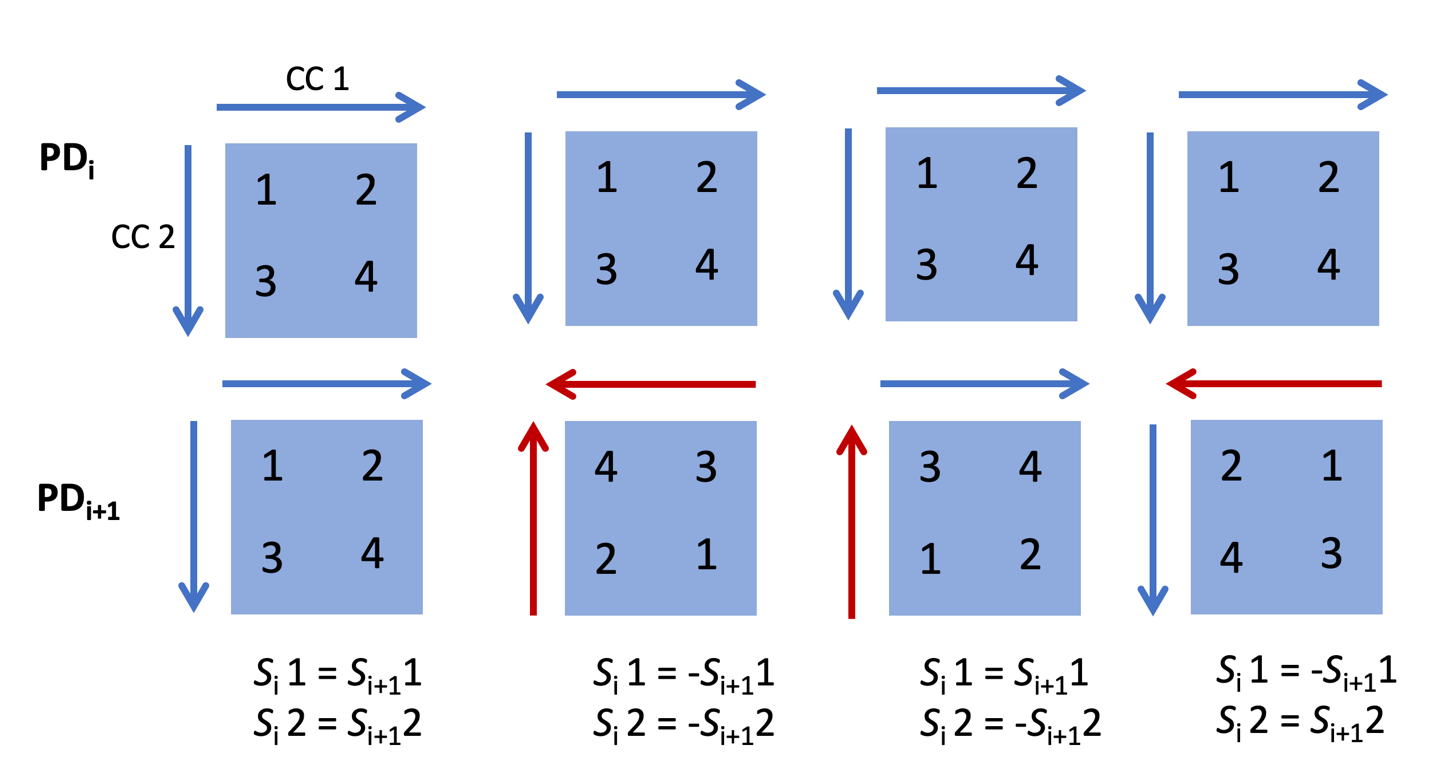
**

**Supplementary Fig. 6.**

**Four schematic representations illustrating the sign change in CC1 and CC2 between two adjacent projection directions.**
